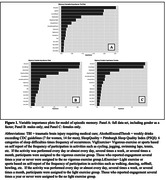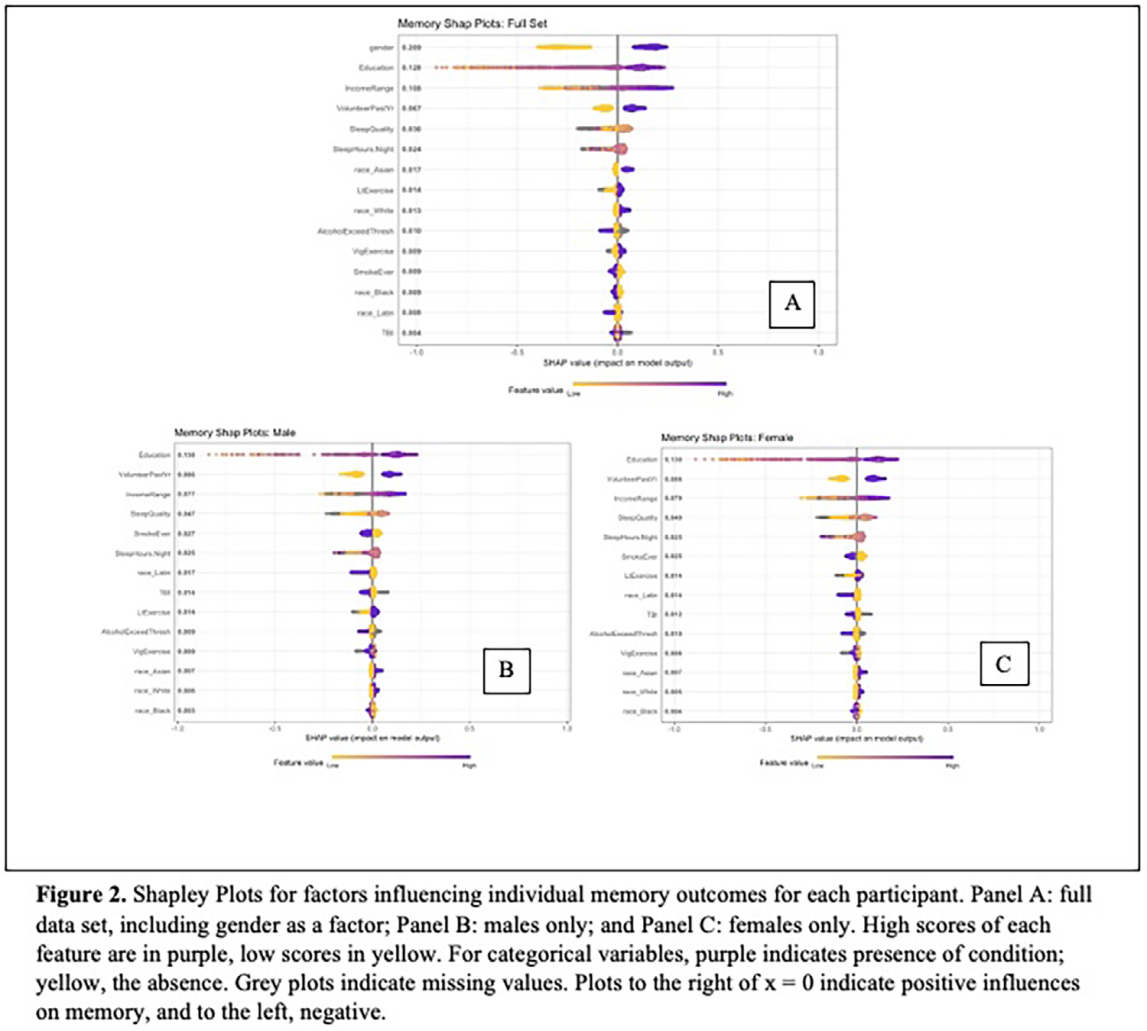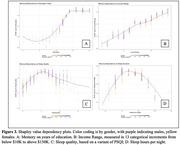# A machine learning approach to multifactorial modeling of episodic memory performance

**DOI:** 10.1002/alz.089749

**Published:** 2025-01-03

**Authors:** Evan Fletcher, Marianne Chanti‐Ketterl, Emily Hokett, Yi Lor, Umesh M Venkatesan, Paola Gilsanz, Rachel A. Whitmer, Ruijia Chen, Kristen M. George, Zvinka Zlatar

**Affiliations:** ^1^ UC Davis, Davis, CA USA; ^2^ Duke‐UNC Alzheimer’s Disease Research Center, Durham, NC USA; ^3^ Columbia University Irving Medical Center, New York, NY USA; ^4^ University of California, Davis, Davis, CA USA; ^5^ Moss Rehabilitation Research Institute, Elkins Park, PA USA; ^6^ Thomas Jefferson University, Philadelphia, PA USA; ^7^ Kaiser Permanente Northern California Division of Research, Oakland, CA USA; ^8^ University of California, Davis School of Medicine, Sacramento, CA USA; ^9^ University of California, San Francisco, San Francisco, CA USA; ^10^ University of California, San Diego, La Jolla, CA USA

## Abstract

**Background:**

Neurocognitive health is influenced by multiple modifiable and non‐modifiable lifestyle factors. Machine learning tools offer a promising approach to better understand complex models of cognitive function. We used extreme gradient boosting (XG Boost), an algorithm of decision‐tree modeling, to analyze the association between 15 late‐life lifestyle and demographic factors with episodic memory performance.

**Method:**

Our dataset consisted of 2247 participants from the KHANDLE and STAR cohorts. Participants included 841 men and 1406 women, an ethnoracial diversity of 413 Asian, 987 Black, 349 Latinx, and 496 White adults with age range 54‐90 (mean = 74). XG Boost models of continuous episodic memory performance were trained and evaluated using a random training/test split of the data. Trained models were used to plot relative factor importance and individual Shapley values for each participant, over the entire sample and stratified by gender.

**Result:**

We present XG boost ranking of factors for episodic memory in the full set and stratified by gender. The relative importance of factors for baseline episodic memory (Fig. 1) showed education, income range and sleep quality as the most important variables in both full and sex‐stratified models. In the full sample, gender was highly important and ethnoracial categories showed the least importance.

Shapley values for each variable (Fig. 2) showed gender, education, income range, volunteering, and sleep quality were paramount contributors to episodic memory performance. In gender‐stratified models, education, volunteering, income, and sleep quality were of most importance. Exercise contributed modestly. Negative factors like smoking, excessive alcohol and TBI had moderate impacts. Ethnoracial background had minimal influence, except for White (full set) and Latinx (male and female).

Shapley dependency on memory in the full set (Fig. 3) showed a plateau effect for education at around 12 years of schooling; linearly increasing effect with no plateau for income; an inverted “U” dependence on sleep duration; and a nonlinear relation with sleep quality requiring more investigation.

**Conclusion:**

Decision trees may be a promising approach to determining the relative contributions of multiple life exposure factors that affect cognitive performance in a large and diverse cohort. This may help to inform ADRD preventive interventions.